# Haplotypes in the Complement Factor H (CFH) Gene: Associations with Drusen and Advanced Age-Related Macular Degeneration

**DOI:** 10.1371/journal.pone.0001197

**Published:** 2007-11-28

**Authors:** Peter J. Francis, Dennis W. Schultz, Sara Hamon, Jurg Ott, Richard G. Weleber, Michael L. Klein

**Affiliations:** 1 Macular Degeneration Center, Casey Eye Institute, Oregon Health & Science University, Portland, Oregon, United States of America; 2 Rockefeller University, New York, New York, United States of America; Rutgers University, United States of America

## Abstract

**Background:**

Age-related macular degeneration (AMD), the leading cause of blindness in the Western world, is a complex disease that affects people over 50 years old. The complement factor H (CFH) gene has been repeatedly shown to be a major factor in determining susceptibility to the advanced form of the condition. We aimed to better understand the functional role of this gene in the AMD disease process and assess whether it is associated with earlier forms of the disease.

**Methodology/Principal Findings:**

We genotyped SNPs at the CFH gene locus in three independent populations with AMD: (a) extended families where at least 3 family members had AMD; (b) sporadic cases of advanced AMD and (c) cases from the Age-Related Eye Disease Study (AREDS). We investigated polymorphisms and haplotypes in and around the CFH gene to assess their role in AMD. CFH is associated with early/intermediate and advanced AMD in both familial and sporadic cases. In our populations, the CFH SNP, rs2274700, is most strongly associated with AMD and when incorporated into a haplotype with the Y402H SNP and rs1061147, the strongest association is observed (p<10^−9^).

**Conclusions/Significance:**

Our results, reproduced in three populations that represent the spectrum of AMD cases, provide evidence that the CFH gene is associated with drusen as well as with advanced AMD. We also identified novel susceptibility and protective haplotypes in the AMD populations.

## Introduction

Age-related macular degeneration (AMD) is the leading cause of blindness in the developed world.[1] The hallmark of AMD is the progressive accumulation of drusen (sub-retinal yellow deposits) at the macula, that portion of the central retina specialized for fine visual tasks. Evidence suggests that the risk of developing advanced complications of the disease correlates with the amount of drusen present in the macula. For instance, the presence of extensive large (>125 µm in minimum diameter) drusen >393,744 µm^2^ in area represented the highest risk category for the development of advanced AMD (19.2% in five years and 55.3% in 10 years) in the Beaver Dam Eye Study.[2]

Advanced AMD is characterized by poor central vision following the development of: (1) choroidal neovascularization (CNV) associated with leakage, hemorrhage, and subretinal scarring, or (2) geographic atrophy (GA), characterized by the appearance of coalescing foci of retinal pigment epithelial atrophy.[1] AMD is a complex disease since its development is determined by the effects and interactions of a number of genes and environmental risk factors. In the last few years, considerable advances have been made in identifying AMD susceptibility genes, in particular those implicated in the development of advanced disease. The association of single nucleotide polymorphisms (SNPs) in the CFH (Y402H, C1277T, rs1061170)[3]–[7] and LOC387715 (A69S, rs10490924)[8]–[11] and HTRA1 genes[12] with advanced AMD susceptibility have now been replicated in a number of independent studies. Furthermore, there is now evidence that specific SNP haplotypes within the chromosomal loci of these genes confer increased risk of CNV development or appear relatively protective.[13], [14]

Other genes have been implicated in AMD development, including complement component 2 (CF2) and factor B (BF),[15] toll-like receptor 4 (TLR4),[16] and potentially a sequence variant in hemicentin-1 (HMCN-1)[17], [18] which resides close to the CFH locus on 1q. However, the precise role of these genes and others[11] in determining susceptibility to the condition remains to be fully evaluated. The role of these genes in earlier AMD phenotypes is not yet established.

In this article, we present the results of analyses of 3 populations with AMD: extended families genetically enriched for their predisposition to the disease, sporadic cases of advanced AMD, and the Age-Related Eye Disease Study (AREDS) cohort. We investigated associations with polymorphisms and haplotypes in the CFH gene. Furthermore, since our cohorts included individuals with earlier stages of AMD, the potential role of CFH in drusen formation was explored.

## Results

### Association of individual SNPs in and around CFH with advanced AMD

The CFH risk allele rs1061170 ‘C’ and AMD was strongly associated with advanced AMD status in each of the three cohorts: (1) a cohort of families with AMD (1253 Caucasian affected and unaffected individuals from 124 extended families (average age 68.2 years; 492 males, 761 females); (2) a sporadic case/control cohort: 211 sporadic cases (age 79 years, range 57–100; 70 male, 141 female; 210 Caucasian, 1 Other racial origin) of advanced AMD and 183 unrelated ophthalmologically evaluated controls (age 74, range 63–92; 81 male, 102 female; 183 Caucasian; 2201 cases and controls from the Age-related Eye Disease Study (AREDS).

Associations with several other SNPs in the CFH gene and advanced AMD status were also surveyed in these populations and significant associations were also noted for SNPs rs529825, rs800292, rs1061147, rs203674 and rs2274700 (table 1). The SNP most strongly associated with advanced AMD was the synonymous exon 10 SNP, rs2274700.

**Table 1:** Single SNP analysis.

SNP	Location and change	Sample	OR (lower bound–upper bound)	P-value	Fbat p value
rs529825	IVS1	Family Data			**0.013**
		Case Control	2.67 (2.22–3.12)	**<0.0001**	
		AREDS	2.57 (1.81–3.33)	0.018	
rs800292	Exon 2, I62V	Family Data			**0.002**
		Case Control	2.35 (1.95–2.75)	**<0.0001**	
		AREDS	2.52 (1.75–3.28)	0.022	
rs3766404	IVS6	Family Data			0.096
		Case Control	3.82 (3.292–4.354)	**<0.0001**	
		AREDS	3.77 (2.59–4.95)	0.030	
rs1061147	Exon 7, A307A	Family Data			**0.004**
		Case Control	2.53 (2.21–2.85)	**<0.0001**	
		AREDS	2.05 (1.54–2.56)	**0.008**	
rs1061170	Exon 9, Y402H	Family Data	4.36 (3.51–5.21)†		**0.006**
		Case Control	2.40 (2.07–2.73)	**<0.0001**	
		AREDS	2.04 (1.54–2.54)	**0.007**	
rs203674	IVS10	Family Data			**0.02**
		Case Control	2.68 (2.34–3.02)	**<0.0001**	
		AREDS	2.13 (1.61–2.65)	**0.006**	
rs2274700	Exon 10, A473A	Family Data			***0.0002***
		Case Control	3.07 (2.74–3.41)	***<0.0001***	
		AREDS	3.32 (2.67–3.97)	***0.0003***	

Emboldened numbers indicate statistically significant p-values allowing for multiple testing. Number in italics indicated most significant p-value. For IVS1 and I62V confer decreased odds of developing AMD.

### Susceptibility and protective haplotypes at the CFH locus

A subtractive approach utilized 27 haplotypes to determine the combination of SNPs with the lowest p-value of association with AMD using FBAT. This best combination comprised the following 3 SNPs: rs1061147, rs1061170, rs2274700 (p<0.0002, Table 2). Two nucleotide combinations of this 3-SNP haplotype were significantly associated with AMD status: rs1061147 ‘A’, rs1061170 ‘C’, rs2274700 ‘C’ and rs1061147 ‘C’, rs1061170 ‘T’, rs2274700 ‘T’. The other rs1061147, rs1061170, rs2274700 haplotype ‘C T C’ was not associated with AMD.

**Table 2:** Haplotypes and their association with AMD in the familial AMD cohort.

Number of SNPs	SNPs involved in best model	Haplotypes	Frequency	Haplotype p-values	Overall p-value
7	rs3766404 rs529825 rs1061147 rs800292 rs1061170 rs203674 rs2274700	TCACCCC	0.58	0.03	
		TCCCTAC	0.12	0.90	
		TTCTTAT	0.11	0.10	0.12
6	rs3766404 rs529825 rs1061147 rs800292 rs1061170 rs2274700	TCACC C	0.56	0.02	
		TCCCT C	0.18	0.44	
		TTCTT T	0.11	0.13	
		CCCCT T	0.06	0.09	0.02
5	rs529825 rs1061147 rs800292 rs1061170 rs2274700	CACC C	0.55	0.01	
		CCCT C	0.18	0.31	
		TCTT T	0.11	0.18	
		CCCT T	0.10	0.02	0.006
4	rs1061147 rs800292 rs1061170 rs2274700	ACC C	0.56	0.00	
		CCT C	0.18	0.31	
		CTT T	0.12	0.07	
		CCT T	0.100	0.03	0.0008
3	*rs1061147 rs1061170 rs2274700*	*A* ***C*** *C*	*0.56*	*0.0006*	
		*C T T*	*0.25*	*0.0003*	
		C T C	0.17	0.35	0.00008
2	rs1061147 rs1061170	A **C**	0.56	0.00008	
		C T	0.41	0.004	0.0001
1	rs1061170	**C**	0.58	0.006	
		T	0.42	0.006	0.006

The most strongly associated haplotype (shown in red) is derived from SNPs genotyped in the CFH gene locus using backward selection. Emboldened ‘C’ indicates CFH rs1061170 risk allele. ‘Best’ haplotype shown in italics. Overall p value describes association of the haplotype with AMD independent of alleles

We then investigated whether these haplotypes were associated with AMD development in the AREDS and case-control populations and found similar results (Table 3). Since the ‘CTC’ haplotype conferred no additional odds of developing AMD, it was also possible in the populations to assess the direction of the effects, finding that the ACC haplotype conferred higher disease odds and the CTT haplotype conferred lower disease odds of advanced AMD.

**Table 3:** Replication of best haplotype in (a) case control data and (b) AREDS data.

Case control cohort							
Haplotype	Hap Score	Pooled Haplotype Frequency	Control Haplotype Frequency	Case Haplotype Frequency	OR (lower bound–upper bound)	Associated P-value	Overall p value
ACC	−5.85	0.47	0.36	0.57	0.43 (0.29–0.64)	<10^−9^	<0.00001
CTT	−7.83	0.33	0.48	0.20	3.88 (2.51–6.01)	<10^−14^	
CTC	−1.07	0.20	0.16	0.23	0.62 (0.38–1.03)	0.28	

AREDS cohort							
Haplotype	Hap Score	Pooled Haplotype Frequency	Control Haplotype Frequency	Case Haplotype Frequency	OR (lower bound–upper bound)	Associated P-value	Overall p value
ACC	−4.28	0.44	0.35	0.58	0.40 (0.30–0.52)	<0.0001	<0.00001
CTT	−4.79	0.36	0.46	0.21	3.08 (2.30–4.13)	<0.0001	
CTC	−0.30	0.20	0.19	0.21	0.90 (0.64–1.27)	0.77	

The odds ratios are computed by comparing each haplotype against all other haplotypes pooled. Cases defined as AREDS category 4, advanced AMD. Controls defined as AREDS category 1, unaffected. Overall p value describes association of the haplotype with AMD independent of alleles

In our 3 populations, we found that neither individual SNPs nor the 3-SNP haplotype was preferentially associated with either the neovascular or geographic atrophy form of advanced AMD.

### Association of CFH (Y402H) with early and intermediate AMD

Table 4 shows the prevalence of rs1061170 (Y402H) for each category of AMD from category 1 (no AMD), through increasing stages of drusen (categories 2 and 3) to advanced AMD (category 4) in both the AREDS and familial AMD cohorts.

**Table 4:** Frequency of the rs1061170 CFH Y402H risk allele ‘C’ by category of AMD.

AMD category	AREDS cohort			Familial AMD cohort			Case control cohort		
	Number of individuals	‘C’ allele frequency	P value	Number of individuals	‘C’ allele frequency	P value	Number of individuals	‘C’ allele frequency	P value
1	306	0.36		97	0.47		211	0.36	
2	502	0.38	0.46	55	0.49	0.84			
3	526	0.48	<10^−5^	95	0.63	0.002			
4	578	0.61	<10^−22^	124	0.63	0.001	183	0.59	<10^−10^

P-values calculated for allelic test of association for differences in AMD grade (categories 2, 3 and 4) from category 1 (no AMD)

The highest prevalence of this risk allele is in advanced AMD (category 4) and lowest in the unaffected controls (category 1, no AMD), as illustrated in Table 4. The prevalence of the risk allele increases in those with mild drusen (category 2, early AMD) and increases further in eyes with large drusen (category 3, intermediate AMD).

## Discussion

In this study, we confirm the strong association between polymorphisms in the complement factor H gene and advanced age-related macular degeneration in 3 independent predominantly Caucasian populations. For the first time, we report this association in families in whom there is increased susceptibility among relatives to develop the condition. The prevalence of the risk allele ‘C’ for the coding SNP rs1061170 (Y402H) present in exon 9 of the gene in cases of advanced AMD is broadly comparable to that previously reported in sporadic case-control populations[3], [4] and in the other 2 cohorts evaluated in this study.[12] Similarly, the odds ratio of having advanced AMD, based on the number of ‘C’ alleles present, was also comparable to previous studies.

We chose the 7 SNPs to be genotyped based upon those previously shown to be associated with AMD at the CFH gene locus: rs529825, rs3766404, rs1061147, rs800292, rs1061170, rs203674 and rs2274700.[4] A backward step-wise selection method and the FBAT were chosen because multi-SNP effects are allowed that are not additive. If a forward selection method is used, those SNPs that have the largest additive effect are most likely to be in the model. If 2 SNPs have a non-additive effect but no large marginal effects, it would be impossible to detect them using forward selection. If 2 SNPs only have an effect simultaneously, using this model misses them. Only if a SNP has a combined influence with SNPs that already have a large effect will it be detected by forward selection. Using backward step-wise selection, we identified a 3-SNP haplotype comprising rs1061147 ‘A’, rs1061170 ‘C’, rs2274700 ‘C’ as the most strongly associated with AMD in our familial AMD cohort. Analyses performed in our other 2 populations comprising sporadic cases and controls and the AREDS cohorts confirmed this association.

There are considerable similarities between the findings in our study of CFH haplotypes and the other three studies previously published on the subject. Hageman and colleagues[4] sequenced all 22 coding exons of the CFH gene and their 50- to 100-bp flanking intronic sequences in the University of Iowa cohort, finding a total of 26 sequence variants: 17 SNPs in the coding region, including 5 synonymous and 12 non-synonymous substitutions and 9 intronic SNPs. They found several highly significant associations with AMD, including the non-synonymous I62V, Y402H and the IVS2 variants. The strongest association with AMD in this cohort was observed with the synonymous A473A (rs2274700) variant in exon 10. In our study of 3 independent populations, we similarly find these associations and show that their most significantly associated variants form 2 of the 3 SNPs in our haplotype. In addition, when the non-disease associated alleles are inherited in this haplotype, they appear protective for the presence of AMD.

Li[14] examined 84 polymorphisms in and around CFH in 726 affected individuals in a case-control cohort, finding 20 polymorphisms that showed stronger association with disease susceptibility than the Y402H variant. These investigators defined a set of 4 common haplotypes (of which 2 were associated with disease susceptibility and 2 appeared protective). Their strongest associated SNP was the synonymous variant rs2274700 (A473A), which is one of the SNPs in our haplotypes that confer increased or decreased risk of AMD.

Maller and coworkers[13] performed a case-control study drawn from a US-based population of European descent, identifying a previously unrecognized common, non-coding variant in CFH (rs1410996) that was associated with advanced AMD as well as the previously recognized Y402H change. Using stepwise logistic regression, even after conditioning for each of the SNPs, the authors found that both remained strongly associated with disease. In our study, we did not genotype for the rs1410996 SNP. However, our 3-SNP disease-associated haplotype does comprise the Y402H sequence change.

In summary, our 3-SNP haplotype is a good ‘fit’ for those previously described. Furthermore, we have been able to confirm its validity in 3 independent well-phenotyped populations. Interestingly, apart from the CFH Y402H SNP, the other two are synonymous coding variants, suggesting that these sequence changes do not combine to effect some reduction in protein function but more likely alter expression levels of the CFH gene and possibly other complement factor-related genes[14].

It is now established that the Y402H CFH change is associated with advanced AMD. Whether this polymorphism also contributes to other earlier AMD phenotypes remains to be fully explored. Only one other report has addressed this issue finding increased frequency of the risk allele in individuals with drusen[19]. Because two of our cohorts (AREDS and familial AMD) comprised individuals with intermediate stages of AMD, ie varying degrees of drusen, in comparable categories, we were able to address this question. There was a statistically significant association with increasing amounts of drusen in both of these cohorts suggesting a role for CFH in these earlier stages of AMD. Such an association is biologically plausible since complement factors, including CFH have been identified in drusen.[20]

We found that the frequency of the risk allele was similar among those with advanced AMD in all three groups (approximately 0.60). Interestingly, this same allele frequency is observed in those individuals with intermediate AMD (drusen) with a strong family history of AMD and higher than in those with the same category of AMD in the AREDS cohort. Our future longitudinal studies will evaluate whether this is because individuals with a family history of AMD, who have both extensive drusen and the CFH risk allele are at very high risk of progression to choroidal neovascularization and/or geographic atrophy.

Previous studies have failed to show that CFH associates more frequently with the presence of either CNV or GA. In our 3 populations, we also found that neither the SNPs in and around the CFH gene nor the 3-SNP haplotype preferentially increased susceptibility to one of these late complications.

In summary, we have examined 3 independent, carefully phenotyped populations with AMD. These populations can be considered to reflect the full diversity of AMD patients: those with familial susceptibility to the condition and 2 case-control populations, one of which was followed for more than 10 years as part of a nationwide clinical trial. We have confirmed the association between the Y402H CFH mutation and advanced AMD finding however the strongest association with another SNP within the gene, rs2274700 in agreement with two other studies[13], [14]. In addition, we have found that these two SNPs, when part of a 3-SNP haplotype confer considerably increased odds of AMD development compared with the SNPs assessed singly. Our data also makes a case for CFH to have a role in drusen formation and progression as well as the development of late complications, such as choroidal neovascularization or geographic atrophy.

## Materials and Methods

All studies involving human subjects were approved by the Institutional Review Board of Oregon Health & Science University. Research followed the tenets of the Declaration of Helsinki, and written informed consent was obtained from all subjects after explanation of the nature and possible consequences of the study.

### Study populations and phenotyping

#### AMD family cohort

Each family comprised at least three examined (and genotyped) affected relatives with AMD. Individuals were identified as follows: category 1, (no AMD) no drusen or drusen less than 63 µm diameter, and no pigment changes in either eye; category 2, mild to moderate drusen consisting of drusen of any size, but <393,744 µm^2^ in total area within 1500 µm from the fovea, with or without pigment changes; category 3, extensive large drusen (>125 µm in minimum diameter) >393,744 µm^2^ in area (a minimum of approximately 20 large drusen) within 1500 µm of the fovea, with or without pigment changes and no evidence of advanced AMD; category 4 (advanced AMD), presence in one or both eyes of advanced macular degeneration (choroidal neovascularization or geographic atrophy)[21], [22]. For the purposes of cross-comparison between cohorts, these categories are similar to AREDS categories 1–4. Categories 2 and 3 are equivalent to AREDS category 3, with category 3 eyes considered an extremely high risk group for progression to advanced AMD (geographic atrophy or choroidal neovascularization).

*Sporadic cases and controls* were ascertained from our clinical practice. Diagnosis of AMD in cases was based upon the presence of geographic atrophy or choroidal neovascularization (equivalent to AREDS category 4, see below).[23] Control subjects were at least 60 years of age, with no signs of AMD defined as no drusen larger than 63 microns in diameter.

#### AREDS cohort

The Age-Related Eye Disease Study was an eleven-center double-masked clinical trial investigating the effects of oral antioxidant and vitamin supplements on disease progression. DNA was extracted from venous blood from 2201 individuals (939 males, 1262 females; 2115 Caucasian, 72 African American, 6 Hispanic, 3 Asian, 5 Other racial origin) which comprised approximately one-half the AREDS participants (obtained from the AREDS Genetic Repository). Participants were aged 67.7 years (range 55–80) years. The AREDS study procedures have been previously reported.[24], [25] Briefly, participants were classified into 5 groups: category 1 (343 individuals), no AMD; category 2 (578 individuals), extensive small drusen or intermediate drusen; category 3 (624 individuals), large drusen, non-central geographic atrophy or pigment abnormalities in 1 or both eyes; category 4 (285 individuals), advanced AMD (CNV or GA) or vision loss due to AMD in one eye. At least one eye had best-corrected visual acuity of 20/32 or better21; category 5 (368 individuals), advanced AMD in both eyes. All patients were included in this study irrespective of their treatment assignment.

### Genotyping

Seven SNPs in and around the CFH gene locus were chosen: rs3766404, rs529825, rs1061147, rs800292, rs1061170, rs203674 and rs2274700. Genotyping was performed using Sanger sequencing at Prevention Genetics (3700 Downwind Drive, Marshfield, Wisconsin, USA) and at the Broad Institute Center for Genotyping and Analysis, Cambridge, MA, USA.

### Statistical analyses

#### Association analyses

To estimate SNP frequencies and determine if the frequencies differed between affected and unaffected individuals in the pedigrees, the Family Based Association Test (FBAT) was used.[26] Adjusted unconditional logistic regression was used to determine the relationship between the CFH rs1061170 genotype and other CFH SNPs and AMD status in the other 2 populations. Additionally, a test for trend related to the number of risk alleles (0, 1, 2) was calculated.

#### Haplotype analyses

FBAT was used to estimate haplotype frequencies and any that occurred less than 10 times in combined cases and controls were eliminated from the analysis. Since 7 SNPs were measured in this gene, all 7 SNPs (see above genotyping) were used to produce the first haplotype model. Then 1 SNP was removed at a time to create 7 models, each containing 6 SNPs. Of these 7 models, the one with the lowest associated p-value was deemed the best model. Then 1 SNP was removed at a time from this best 6-SNP model to create 6 models, each containing 5 SNPs. The 5-SNP model with the lowest associated p-value was then the best 5-SNP model. This process was continued to find the best 4-SNP, 3-SNP, 2-SNP and 1-SNP models. Overall, we performed 27 tests. All 27 models were evaluated to determine which had the lowest associated p-value. The Bonferroni was used to adjust for multiple testing.

The SNPs from the model with the lowest associated p-value were used to determine if one haplotype effect replicated in 2 additional samples. We used haplo.stat (a program in R) to estimate haplotype frequencies, determine if these differed between cases and controls, determine if individual haplotypes differed significantly between cases and controls and estimate an odds ratio for each individual haplotype.[27] Any of the haplotypes that occurred less than 10 times in combined cases and controls were eliminated from the analysis.
